# First report, morphological and molecular characterization of *Xiphinema elongatum* and *X. pachtaicum* (Nematoda, Longidoridae) from Ethiopia

**DOI:** 10.3897/zookeys.489.8629

**Published:** 2015-02-18

**Authors:** Gezahegne Getaneh, Wim Bert, Wilfrida Decraemer

**Affiliations:** 1 College of Veterinary Medicine and Agriculture, Addis Ababa University, Salale Campus, P.O.Box 245, Fitche, Ethiopia; 2 Department of Biology, Ghent University, Ledeganckstraat 35, 9000 Ghent, Belgium

**Keywords:** 18S rDNA, Ethiopia, molecular data, morphometry, phylogeny, *Xiphinema*

## Abstract

A total of six soil samples were collected around rhizosphere of citrus plants during 2010 from Melkassa Agricultural Research Center experimental station, Ethiopia. From these samples two most important ecto-plant parasitic nematodes of the genus *Xiphinema* were found and analysed. The genus *Xiphinema* is a large group of the phylum nematoda which constitutes more than 260 species. They are polyphagous root- ectoparasites of many crop plants and some species of this genus cause damage by direct feeding on root tips and transmit nepoviruses. The delimitation and discrimination of two species in the genus is presented, described herein as *Xiphinema elongatum* and *Xiphinema pachtaicum*. Morphological and morphometric data were done using light microscopy and results of both species were fit within the previously described nematode species of *Xiphinema elongatum* and *Xiphinema pachtaicum*. 18S rDNA were analysed using Bayesian inference (BI) method to reconstruct phylogenetic relationships of the studied *Xiphinema* sp. (KP407872 *Xiphinema elongatum* and KP407873 *Xiphinema pachtaicum*) with other *Xiphinema* species. The 18S rDNA sequence of *Xiphinema pachtaicum* was alike to previously described species from the GenBank but *Xiphinema elongatum* exhibited very small levels of nucleotides differences (0.4%) which might be possible intra-specific divergence. Though this region of rDNA has less resolution on complex species, its combination with morphological and morphometric analyses, suggests these species as *Xiphinema elongatum* and *Xiphinema pachtaicum* with the GenBank accession number of KP407872 and KP407873, respectively. Short notes, morphological measurements, illustrations, and molecular data are given to these species. These species are reported for the first time from Ethiopia and it provides new geographical information of these organisms.

## Introduction

The ecto-parasitic longidorid nematodes of the genus *Xiphinema* is amongst the ten most economically important plant parasitic nematode genera (Sasser and Freckman 1987). They are migratory and polyphagous nematodes, which cause damage to a broad range of crop plants by their direct feeding on root tips which results in root gall and stunted shoot growth. Approximately 4% of *Xiphinema* species have been shown to transmit certain nepoviruses to a wide range of fruit and vegetable crops (Taylor and Brown 1997). *Xiphinema* is the largest genus of the phylum Nematoda (Andrassy 2007) and currently has more than 260 valid species, of which approximately 50 species belong to the *Xiphinema americanum* group (Gutiérrez-Gutiérrez et al. 2013; Oliveira and Neilson 2006). Because of their economic importance, species of the *Xiphinema americanum* group are listed as A1 quarantine organisms by European and Mediterranean plant protection organization (EPPO 2011; Decraemer and Robbins 2007).

The genus *Xiphinema* has characteristic morphological features of 1.2–7.3 mm body length, flanged odontophore, forked junction of the odontostyle and odontophore, posterior strongly sclerotized and slightly sclerotized anterior border of the double guiding ring near the odontostyle/odontophore junction. Amphid fovea, mainly funnel- or stirrup shaped with aperture slit like and dorsal pharyngeal gland nucleus close to dorsal gland opening (Hunt 1993).

Accurate identification of this nematode is needed to allow distinction between virus vector and non-virus vector species which helps to differentiate species under quarantine or regulatory strategies. *Xiphinema* species show quite some morphological complexity to identify only based on morphological identification method (Lamberti et al. 2000; Kumari et al. 2010). Ribosomal DNA (rDNA) sequences from partial 18S, ITS regions, and the D2 and D3 expansion segments of the 28S, and mitochondrial DNA (mtDNA), cytochrome c oxidase 1 subunit (COI), are useful diagnostic tool for the characterization and establishment of phylogenetic relationships especially for the species of the *Xiphinema americanum* group (Lazarova et al. 2006; Gutierrez-Gutierrez et al. 2010). Though the sequence of rDNA of partial 18S sequence is considered as lesser importance for species delimitation, it is used for phylogenetic characterization of some species of the genus. In this study, the 18S small subunit of the rDNA region was analysed.

Both samples were collected from main horticultural crop production fields (mainly from citrus plantation) in the Rift valley basin of Ethiopia. Two *Xiphinema* species, *Xiphinema elongatum* Schuurmans Stekhoven & Teunissen, 1939 and *Xiphinema pachtaicum* (Tulaganov, 1939) were found and are herewith described using morphology, morphometric data and molecular phylogenetic analyses. These findings represent new records from Ethiopia as well it represents new geographical information.

## Materials and methods

### Sampling and morphological observations

Samples of both species were taken from rhizosphere of citrus plants in the Rift valley basin of Ethiopia (39°21'E, 8°24'N) in 2010. A total of six bulk samples of each 1–1.5 kg consisted of 10–15 cores taken from the top 10–40 cm of soil. The samples were kept cool in plastic bags during transportation to the laboratory for nematode extraction. Nematodes were extracted from 200g soil of sub-samples using the modified Baermann method (Hooper 1985).

Specimens were fixed by 4% formalin with 1% glycerin that heated to 70 °C and added quickly to kill and fix nematodes in one step (Seinhorst 1966). The fixed specimens were processed to anhydrous glycerin following the glycerin-ethanol method of Seinhorst (1959) modified by De Grisse (1969). Fixed specimens were permanently mounted in anhydrous glycerol (Hooper 1985). For morphological study, specimens were photographed using Olympus BX50 and Olympus CH30 light microscopes. Voucher specimens for *Xiphinema elongatum* were deposited at Ghent University nematode collection as UGnem-37 and specimens of *Xiphinema pachtaicum* were placed at Ambo plant protection Research Center Nematology section, Ethiopian Institute of Agricultural Research, Ethiopia.

### DNA extraction, PCR, and sequencing aseptic

Nematode specimens from the same population were also killed and preserved in DESS solution containing 20% dimethyl sulphoxide (DMSO) and 0.25 M disodium EDTA, saturated with NaCl, pH 8.0 (Yoder et al. 2006; Seutin et al. 1991). This was done by pouring the nematode suspension over a 500 mesh sieve (25 μm opening) to allow most of the water to drain and rinsing the nematodes with DESS solution into a vial (Yoder et al. 2006). Individual nematodes from the solution were mounted on temporary slides and identified using light microscope before further molecular characterization of the small subunit (SSU, 18S). These morphologically characterized DESS-preserved nematodes were rinsed in distilled water for about 30 minutes, and transferred to eppendorf tube with 25 µl of worm lysis buffer (WLB), Williams et al. (1992): 50 mM KCl; 10 mM Tris-Cl pH 8.3; 2.5 mM MgCl2; 0.45% NP 40 (Tergitol Sigma); and 0.45% Tween 20) and frozen at -80 °C for at least 10 minutes. To each tube it was added 1 µl of proteinase K (60 µg ml^-1^) prior to incubation at 65 °C for 1 hour followed by enzyme deactivation at 95 °C for 10 minutes. To amplify the 18S region, 2.5 µl of gDNA suspension was used as template in a 25 µl PCR reaction mix (TopTaq Qiagen, Germany) following the manufacturer’s protocol. The primers used were G18S4 (5’- GCT TGT CTC AAA GAT TAA GCC - 3’) & 4F (5’-CAA GGA CGA WAG TTW GAG G-3’) and the reverse primers were 18P (5’- TGA TCC WRC RGC AGG TTC AC - 3’), & 4R (5’- GTA TCT GAT CGC CKT CGA WC-3’) (Blaxter et al. 1998). The PCR conditions were: denaturation at 96 °C for 4 min; followed by 40 cycles of 95 °C for 30 second, 54 °C for 30 second, 72 °C for 1 min, and extension for 10 min at 72 °C. Aliquots of 5 µl of the PCR products were sized with low DNA mass ladder and separated by electrophoresis in 1% agarose gel stained with ethidium bromide and observed under UV Trans-illuminator BioDoc-It Imaging System. The sizes of the amplified products were determined by comparison with DNA ladder. PCR products were enzyme-purified using 1 µl of Exonuclease I + FastAP Thermo-sensitive Alkaline Phosphatase. Purification was done by incubating the mixture for 15 minutes at 37 °C followed by 15 minutes at 85 °C to inactivate enzymes. Cleaned PCR products were then used for cycle sequencing using the ABI Prism BigDye V3.1 Terminator Cycle Sequencing kit following the manufacturer’s protocol. Primers used for sequencing were, 9FX (5’-AAG TCT GGT GCC AGC AGC CGC-3’), 2FX (5’-GGA AGG GCA CCA CCA GGA GTG G-3’), 13R (5’-GGG CAT CAC AGA CCT GTT A-3’), 23F (5’-ATT CCG ATA ACG AGC GAG A-3’), 9R (5’-AGC TGG AAT TAC CGC GGC TG-3’), 26R (5’- CAT TCT TGG CAA ATG CTT TCG-3’) (Blaxter et al. 1998; Meldal et al. 2007). Sequencing was performed in both directions. Both nucleotide sequences are deposited in the GenBank (NCBI) as KP407872 for *Xiphinema elongatum* and KP407873 for *Xiphinema Pachtaicum*.

### Phylogenetic analyses

For phylogenetic analysis, the sequences were aligned with related sequences from GenBank, using ClustalW (Thompson et al. 1994) provided by BioEdit sequence alignment editor (Hall 1999). Phylogenetic analyses were performed by Bayesian inference (BI) method with MrBayes v3.1.2 (Ronquist and Huelsenbeck 2003). A general time-reversible model with rate variation across sites and a proportion of invariable sites (GTR + I + G) was used. Analyses were run for 3 × 106 generations and trees were generated using the last 1,000,000 generations well beyond the burn-in value. Also other methods (maximum parsimony, neighbor joining, maximum likelihood) using PAUP* (Phylogenetic Analysis Using Parsimony) (Swofford 2002) provided the same tree topologies but are not further discussed herein.

## Results and discussions

### Xiphinema elongatum

Taxon classificationAnimaliaDorylaimidaLongidoridae

Schuurmans Stekhoven & Teunissen, 1938

Figure 1 ; Table 1

#### Description.

**Figure 1.**
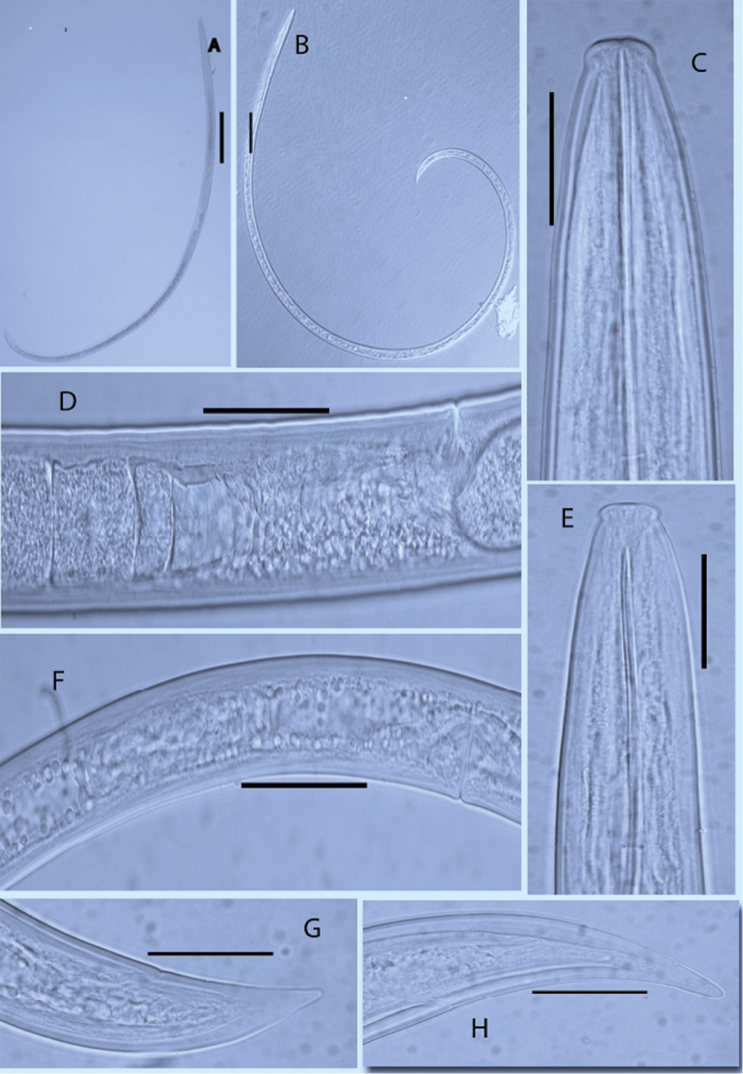
Photomicrographs of *Xiphinema elongatum* and *Xiphinema pachtaicum*. **A, C, D, H** Body habitus, head region, entire female reproductive part, tail region of *Xiphinema elongatum* respectively **B, E, F, G** Body habitus, head region; female reproductive part and tail part of *Xiphinema pachtaicum* respectively. **A** = 250 µm; **B** = 100 µm; **D, F** = 50 µm; **C, E, G, H** = 25 µm

*Female.* Body ‘J’ shaped, cylindrical, tapering towards the anterior end but more to the posterior end. Cuticle smooth, 1.6–2.3 μm thick at neck region, 2.3–3.1 μm at mid body and 5.5–6.3 μm at tail region. Lip region, well demarcated. Amphidial aperture on lip region 50–59% of lip width. Amphidial fovea stirrup–shaped. Guiding ring, about 1/4^th^ of the total odontostyle length from the base of odontostyle. Odontostyle, 1.6 μm diameter, 66±3 (63–73) % of total stylet length and furcated at base. Odontophore well developed with prominent basal flanges 10.9–11.7 μm wide. Lip width 3.1 ±0.5 (2.5–4.2) % of total stylet length. Female reproductive system amphidelphic, didelphic, branches equally developed. Ovaries reflexed. *Pars dilatata oviductus* separated from the uterus by a very robust sphincter muscle. No uterus differentiation. Vagina about half body width and perpendicular to the body axis. Vulva, 41% of body length from anterior end. Tail, conoid to dorsally convex conoid, non-digitate, terminal hyaline portion about 27% of tail length (Fig. 1).

The description of *Xiphinema elongatum* has been recorded by a number of authors and well studied. It was originally described by Schuurmans Stekhoven and Teunissen (1938) from a single female specimen from Rutshuru (Zaire) and redescribed by many authors such as Tarjan and Luc (1963), Williams (1959), Carvalho (1962), Timm (1965), Cohn and Sher (1972), Loof and Maas (1972), Heyns (1974), Williams and Luc (1977) and Loof and Sharma (1979), and also lately by Loof and Luc (1990).

The morphometric data of described Ethiopian specimens were perfectly fit within the twenty-two populations of *Xiphinema elongatum* recorded by Luc and Southey (1980) from a different country and are fairly similar to records of *Xiphinema elongatum* from Botswana (Heyns and Coomans 1991), Guiana and Martinique (Luc and Coomans 1992) and Taiwan (Chen et al. 2004). According to Luc and Southey (1980), *Xiphinema elongatum* appears to have continuous pattern of variation for some morphometric data and shape of tail over different populations of different geographic location. These authors divided *Xiphinema elongatum* into two groups upon morphological variation over different geographical locations. The first group, characterized by a shorter tail and longer stylet, all originate from West Africa whereas the second, having a longer tail and shorter stylet, are mainly from east Africa or South East Asia/ Pacific area. According to this suggestion the studied specimen best fit with the second population group of *Xiphinema elongatum*. This species was reported as widespread and common in Africa including neighbor country Kenya (Luc and Southey 1980; Heyns and Coomans 1991; Coomans et al. 2001).

Accordingly, it belongs to group 7 of the species group, characterized by equal female genital branches, without uterine differentiation, and tail elongate to conical.

As the revised polytomous key by Loof and Luc (1990), on note 22, *Xiphinema elongatum* cannot be separated clearly by the characters used in the key. But they can be differentiated by: c’ = 1.9–3.3; total spear length = 134–178 μm which perfectly fit with the studied Ethiopian specimen.

*Male.* Not found.

**Table 1.** Alpha-numeric codes of the polytomous identification key for *Xiphinema* species by Loof and Luc (1990) of *Xiphinema elongatum* and the studied specimens.

Characters	A	B	C	D	E	F	G	H	I	J	K	L
The studied specimen	4	4	2	3	34	2	1	3	3	-	-	1
Loof and Luc (1990) key	4	4	23	34	2345	23	12	2	3	2	2	1

**Table 2.** Morphometric measurements of *Xiphinema elongatum* and *Xiphinema pachtaicum*. All measurements are in µm, measurements presented as mean ± standard deviation (range).

	*Xiphinema elongatum* (♀)	*Xiphinema pachtaicum* (♀)
n	4	12
L	2380±71 (2330–2430)	1937±103 (1732–2096)
a	69±7 (64–74)	70±3.6 (64–75)
b	7.2±0.5 (6.9–7.6)	7.3±0.6 (6.4–8.2)
c	33.2±5.8 (29.1–37.3)	63±5.1(55–71)
c’	2.7±0.1 (2.6–2.8)	1.9±0.2 (1.6–2.2)
V	41.3 ± 0.3 (41–41.7)	57±1 (56–58)
Lip width	11±1 (10–11)	8±1 (7–10)
Odontostyle	78±12 (70–86)	89±3 (85–97)
Odontophore	51±7 (46–55)	46±5 (33–51)
Pharynx	329±11 (321–337)	266±25 (232–329)
Body width	35±2 (33–36)	28±2 (25–30)
Anal body width	22±3 (20–24)	17±1 (15–18)
Tail	65.1±2.7 (62.5–68)	31±2 (27–36)

#### Locality and host.

The sample materials were collected around the rhizosphere of citrus plant from Melkassa agricultural research center, Oromiya, Ethiopia.

### Xiphinema pachtaicum

Taxon classificationAnimaliaDorylaimidaLongidoridae

(Tulaganov, 1938)

Table 1

Longidorus pachtaicus Tulaganov, 1938: Tulaganov 1938.
Xiphinema pachtaicum (Tulaganov, 1938): Kirjanova 1951.
Xiphinema mediterraneum Martelli & Lamberti, 1967: Siddiqi and Lamberti 1977.
Xiphinema neoelongatum Bajaj & Jairajpuri, 1977: Luc et al. 1984.

#### Description.

*Female.* Body ‘C’ shaped after fixation, tapering to both end but more to the anterior. Cuticle smooth under light microscope. Lip region, distinctly offset by constriction. Amphid aperture post labial, fovea stirrup shaped and about two-third of lip width. Odontostyle robust, poorly forked, 1.56 μm thick, 66±3 (63–73) % of total stylet length and odontophore with weak flanges with width of 10±3 (7–12) μm. Basal Guiding ring 110±6 (104–115) μm from anterior end. Pharynx includes one anterior dorsal nucleus and two posterior subventral nuclei, pharyngeal gland length 94±5 (91–99) μm. Vulva, posterior to mid-body, a transverse slit in ventral view, one-third of the corresponding body width. Female genital branches, didelphic, reflexed, equally developed, generally short. Ovaries, with bacterial endosymbiont, uterus without Z-differentiation, sphincter not clear. Tail short, conical with narrow rounded end (Fig. 1).

Morphological variations of *Xiphinema pachtaicum* have been recorded among populations of different localities from Iran (Fadaei et al. 2003) and Czech (Kumari 2004).

The morphometric range of studied Ethiopian specimen is more similar to that of the Iranian population (Fadaei et al. 2003), and also agrees with the record from Serbia and Montenegro (Basri and Lamberti 2002). The studied Ethiopian species have a slightly longer body length and higher ‘a’ ratio compared to studied population from Iran and Czech (Fadaei et al. 2003; Kumari 2004). However, according to Luc et al. (1984) the variation of coefficient of ‘a’ and ‘c’ are common for this species that is between 43–74 and 47–84 respectively.

*Xiphinema pachtaicum* is widespread in Europe (Switzerland, Germany, United Kingdom, Czech Republic, Slovakia, Hungary, Croatia, Romania, Serbia, Macedonia, Montenegro, Bulgaria, Portugal, Spain, France, Italy, Greece, Cyprus, Malta, Moldova, Ukraine); Asia (Israel, Turkey, Georgia, Uzbekistan, Turkmenistan, Jordan, Iraq, Iran); Africa (Algeria, Morocco, Libya, Egypt, South Africa); North America (United States, Trinidad); South America (Chile) and Australia. This species has not been recorded as a vector of plant viruses (Andrassy 2006).

The alpha-numeric polytomous identification key codes as developed by Lamberti et al. (2000) to be applied for the studied *Xiphinema pachtaicum* of the *Xiphinema americanum* group in Africa are agree with Ethiopian studied population: A 2, B 2, C 1/2, D 32, E 32, F 2, G 21, H 23, I 23, J 1. Characterized by lip region set off from body, body length 1.6 to 2.0 mm; odontostyle length < 86 µm; value of c’ ratio 1.6 to 2; vulva 53 to 56% or vulva > 56%; value of ‘a’ ratio 61 to 80; value of ‘c’ ratio < 60 or > 60; distance of basal guide ring from oral aperture 61 to 75 µm; distance of basal guide ring from oral aperture > 75 µm.

*Male.* Not found.

#### Locality and host.

The sample materials were collected around the root rhizosphere of citrus plant from Melkassa agricultural research center, Oromiya, Ethiopia.

### Molecular and phylogenetic characterization

**Figure 2.**
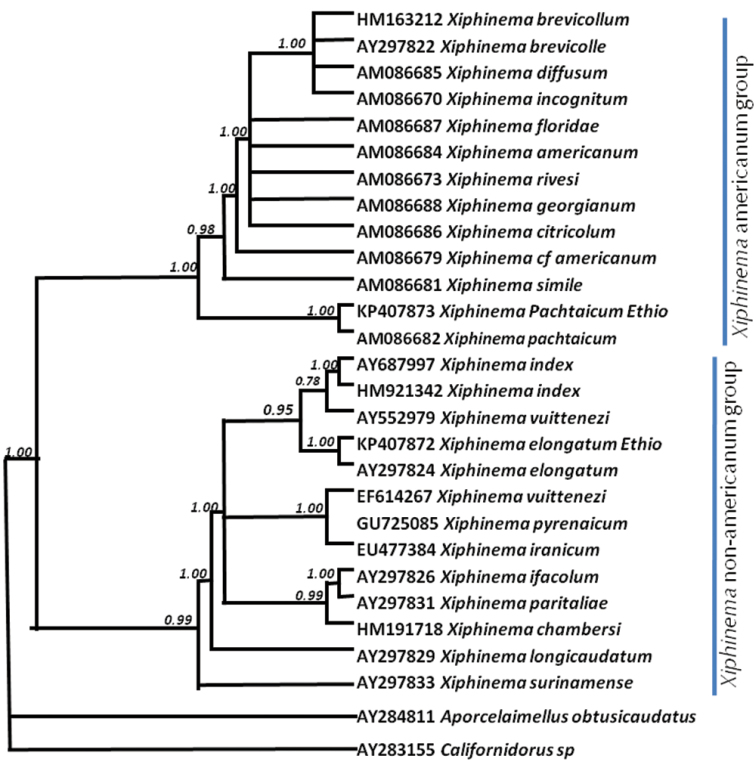
Phylogenetic relationships within *Xiphinema* species by Bayesian 50% majority rule consensus trees as inferred from 18S rRNA gene sequence alignments under the GTR + I + G model.

The PCR amplification of 18S SSU rDNA region of target nematodes with a universal primer were successfully amplified and yielded a single fragment of 1786 bp of *Xiphinema elongatum* species and 1790 bp of *Xiphinema pachtaicum* species. A phylogenetic analysis based on 18S rDNA sequences yielded a well-resolved phylogenetic tree (Fig. 2). This analysis clearly separates the lineage of *Xiphinema americanum* group from the rest of the *Xiphinema* species (Gutierrez-Gutierrez et al. 2010) with maximal support. In this study, the *Xiphinema elongatum* (KP407872) from Ethiopia is grouped with maximal support with *Xiphinema elongatum* AY297824 which was submitted from Brazil (Oliveira et al. 2004). However, 7 bp nucleotide differences (0.4%) were observed between the two populations which could be intraspecific variation between different geographical locations. The studied *Xiphinema pachtaicum* (KP407873) and the Slovakian isolate *Xiphinema pachtaicum* AM086682 had identical sequences. The phylogeny analysis of *Xiphinema pachtaicum* from Spain by Gutierrez-Gutierrez et al. (2011) did not include sequence from 18S region of rDNA and cannot be compared as they analysed the ITS region.

The topology of the tree by other regions of rDNA and position of taxa agrees with previously phylogenetic analysis based on SSU rDNA by van Megen et al. (2009) and Meldal et al. (2007).

This information combined with morphological data can assure the species identity and provide new information on the geographical distribution of the genus *Xiphinema*.

This is the first intensive study on the genus *Xiphinema* from Ethiopia using both morphological and molecular analysis. The morphometric values of *Xiphinema elongatum* and *Xiphinema pachtaicum* described from Ethiopia were similar to previously described species with slight difference in both species in ‘a’ values, but they agree with the range of the population previously recorded by Luc and Southey (1980) and Luc et al. (1984) respectively. Identification of *Xiphinema* species is difficult due to overlapping of many characteristics and their plasticity. Hence, the combination of morphology, morphometric, and molecular results can provide reliable identifications. Based on the congruence of morphological analyses and a SSU rDNA based molecular phylogeny, the Ethiopian *Xiphinema* species were identified as *Xiphinema elongatum* and *Xiphinema pachtaicum*.

## Supplementary Material

XML Treatment for Xiphinema elongatum

XML Treatment for Xiphinema pachtaicum
